# Embedded Oxidized Ag–Pd–Cu Ultrathin Metal Alloy Film Prepared at Low Temperature with Excellent Electronic, Optical, and Mechanical Properties

**DOI:** 10.1021/acsami.1c23766

**Published:** 2022-03-22

**Authors:** Seohan Kim, José Montero, Janghee Yoon, Yunju Choi, Sungmin Park, Pungkeun Song, Lars Österlund

**Affiliations:** † Material Technology Research Institute, Pusan National University, Busan 46241, Korea; ‡ Department of Materials Science and Engineering, The Ångström Laboratory, Uppsala University, P.O. Box 35, SE-75103 Uppsala, Sweden; § Busan Center, Korea Basic Science Institute, Busan 46742, Korea; ∥ Department of Materials Science and Engineering, Pusan National University, Busan 46241, Korea

**Keywords:** transparent conducting materials, ultrathin metal layers, Ag−Pd−Cu alloy, OMO structure, magnetron sputtering

## Abstract

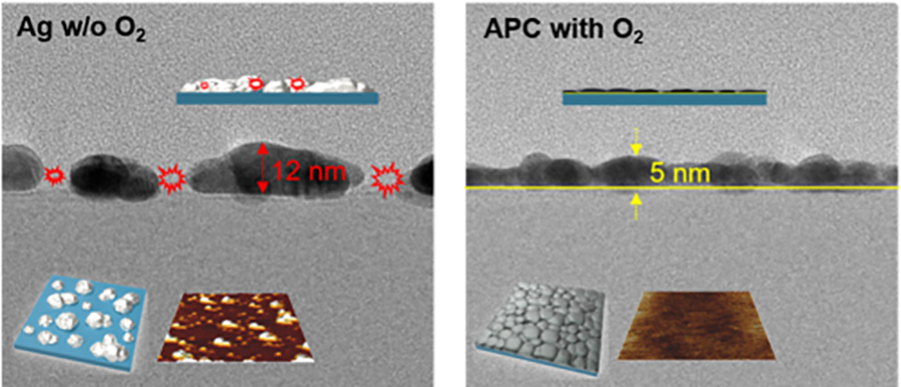

Most transparent conducting materials are based on Sn:In_2_O_3_ (ITO). When applied onto flexible substrates, ITO can be prepared in an oxide–metal–oxide (OMO) configuration, typically ITO/Ag/ITO, where the ductility of the embedded metal layer is intended to reduce the mechanical brittleness and improve the electrical conductivity of the OMO multilayer. Hitherto, the lower limit of the thickness of the Ag layer has been limited by the percolation threshold, which limits the Ag layer to be thicker than ∼10 nm to avoid agglomeration and to ensure conductivity and structural stability. Metal layers of thicknesses below 10 nm are, however, desirable for obtaining OMO coatings with better optical properties. It is known that agglomeration of the metal layer can, to some extent, be suppressed when substituting Ag by an Ag–Pd–Cu (APC) alloy. APC-based OMO films exhibit excellent optical and electrical properties, but still continuous APC films well below 10 nm thickness cannot be achieved. In this work we demonstrate that controlled oxidation of APC results in smooth, ultrathin APC:O continuous coatings (of thickness ∼5 nm) on ITO-coated PET substrates. Moderate oxidation yields superficial PdO_*x*_ formation, which suppresses Ag agglomeration, while still maintaining excellent conductivity. On the other hand, extensive oxidation of APC leads to extensive Pd oxide nucleation deteriorating the conductivity of the film. The ITO/APC:O/ITO films exhibit low resistivity, attributed to a high Hall mobility associated with suppressed agglomeration, good stability in high humidity/temperature environments, superior transmittance in the visible and infrared region, and excellent mechanical bending properties, thus providing new opportunities for fabricating superior transparent conducting coatings on polymer substrates.

## 1 Introduction

Transparent conducting materials (TCM), which as their name implies exhibit high transparency in the visible region and high electrical conductivity, present a multitude of technological applications, most notably in the fields of optoelectronics (displays and touch screen technology)^1−4^ and energy-efficient fenestration.^5^ Most commonly TCMs are based on tin-doped indium oxide In_2_O_3_:Sn (ITO) thin films. Consequently, ITO has been the subject of intensive research during the past decades.^6−8^ However, ITO-based TCMs present some disadvantages, especially when it comes to their implementation in flexible devices.^9−11^ A single ITO thin film suffers from mechanical brittleness and poor electrical stability when deposited onto flexible substrates. Alternatives to ITO for flexible TCMs include metal nanowires,^9,11^ carbon nanotubes,^12−14^ metal meshes,^9,15^ conductive polymers^16^ and, notably, oxide–metal–oxide (OMO) multilayered electrodes,^4,17−21^ where the latter are considered state-of-the-art in the field. The ductility of the metal layer provides mechanical stability to the OMO structure, especially when compared to an equivalent single oxide layer, such as ITO. OMOs present several advantages when compared to other alternatives. In particular, OMO coatings can be easily fabricated by scalable methods onto flexible substrates, including in-line magnetron sputtering, which enables roll-to-roll production.^17,18,22^ The typical OMO structure consists of a thin metal layer (of thickness ranging between 10 and 15 nm) placed between two transparent oxide thin films (40–50 nm thick). The thicknesses of the top and bottom oxide films are selected to achieve optimum optical and electrical properties, whereas the metal layer has to be kept as thin as possible to maintain good transparency. The latter has to be achieved without imperiling the formation of a continuous metal film structure; otherwise, the electrical conductivity of the OMO structure will be adversely affected.

The most common material used as metal layer in the typical OMO structure is Ag. This is due to the excellent electrical conductivity and relatively low cost of Ag when compared to other noble metal alternatives such as Au or Pt. Unfortunately, continuous Ag films below 10 nm are typically not achievable. Examples of OMO multilayered electrodes including Ag as metallic layer are TiO_2_/Ag/TiO_2_,^21^ ITO/Ag/ITO,^18,20^ IZO/Ag/IZO,^19^ and ZnO/Ag/ZnO.^23^

Apart from limited optical transparency in thicker films, the use of Ag as metal layer in an OMO structure presents some problems, in particular when exposed to humid environments. Ross^24^ reported agglomeration of Ag in the ZnO/Ag/ZnO structure after exposure to 95% relative humidity air at room temperature. Aoshima et al.^100^ reported not only the appearance of white spots in a ZnO/Ag/ZnO multilayer after exposure to air at 50 °C and 90% relative humidity but also how the formation of these white spots could be reduced by using an Ag alloy. Thus, Ag–Pd–Cu (APC) alloy films has gained interest as an attractive alternative to pure Ag coatings due to their high electrical stability and resilience toward agglomeration, even when subjected to high relative humidity environments.^4,26^ Jeong et al.^27^ demonstrated that an APC alloy (Pd and Cu content of 0.9 and Cu 1.7 at. %, respectively) exhibited superior resistance to agglomeration and excellent adhesion to the substrate. In addition, Kim et al.^28^ showed how an APC layer placed between bottom and top oxide layers can efficiently reduce diffusion of Ag atoms. Even tough APC in OMO structure has been studied in the past,^4,23,28,25^ there are only few studies focusing on the threshold thickness, i.e., the thickness limit below the percolation threshold or, in other words, the lower limit at which metal *islands* rather than a continuous metallic film is achieved.^22^

In this work, we show that controlled addition of oxygen, in a reactive magnetron process, enables the fabrication of smooth, ultrathin 5 nm thick continuous APC:O films. Such APC:O films exhibit both high electrical conductivity and superior optical properties. Using a low-temperature process, we fabricate OMO films in the configuration ITO/APC:O/ITO, which compare favorably with state-of-the-art OMO structures prepared at high temperatures. The ITO/APC:O/ITO films exhibit high optical transmittance in both visible and near-infrared regions, together with low electrical sheet resistance, and superior mechanical bending performance. The sustained electrical stability of the APC:O-based films was tested in a high-humidity/high-temperature environment (95% relatively humidity and 90 °C) up to 330 h. The results indicate that oxygen incorporated in the ultrathin APC layer provides additional electrical stability with maintained excellent optical properties, which suggests that ITO/APC:O/ITO can be applied as TCMs in a wide range of applications.

## 2 Experimental Details

### 2.1 OMO Fabrication

The ITO/APC:O/ITO multilayer films were prepared by magnetron sputtering onto glass, silicon, and poly(ethylene terephthalate) (PET) flexible substrates following a three-step procedure: (i) deposition of ITO film on a substrate, (ii) deposition of an APC:O layer on the ITO-covered PET substrate, and finally (iii) deposition of the top ITO coating. For steps i and iii, an ITO ceramic target (SnO_2_: 9.8 wt %) was used, while for step ii a target consisting of an APC alloy (Ag: 98 wt %; Pd: 1 wt %; Cu: 1 wt %) was employed and presputtering conducted on ITO and APC targets prior to the deposition process. The DC power for APC deposition was kept low at 30 W by using a calibrated deposition rate of 1 nm/s. Before starting the deposition sequence, the sputtering chamber was evacuated to a base pressure of 1.0 × 10^–6^ Torr. All films (ITOs and APC) were deposited without applying substrate heating. Only Ar gas (>99.9999% purity) was introduced in the chamber during steps i and iii, while for step ii a fractional mixing, Γ, of Ar and O_2_ (>99.999% purity) was employed. Γ is defined (in unit of percentage) as Γ = [ϕ_O_2__/(ϕ_O_2__ + ϕ_Ar_)] × 100, where ϕ_O_2__ and ϕ_Ar_ are the oxygen and argon gas flow introduced in the chamber, respectively. The working pressure in steps i, ii, and iii was set to 5.0, 7.5, and 5.0 mTorr, respectively. Different set of samples were obtained by varying Γ in step ii from 0 to 15%.

### 2.2 OMO Characterization

The electrical and optical properties of the samples were evaluated in a Hall-effect measurement system ECOPIA HMS-3000 and in a Shimadzu UV-1800 spectrophotometer, respectively. The microstructure was determined by X-ray diffraction XRD analysis using a Bruker D8-Advance instrument employing Cu Kα radiation (λ = 1.5412 Å). The surface roughness was estimated by AFM microscopy employing a JPK Nanowizard II instrument. The oxygen content in the thin film was estimated by time-of-flight secondary ion mass spectrometry (TOF-SIMS, ION-TOF GmbH, Münster) using a pulsed 30 keV Bi^+^ primary beam with a current of 1.01 pA, analyzing an area of 200 × 200 μm^2^. The film morphology was monitored by field-emission transmission electron microscopy (FE-TEM, JEOL) operated at a working voltage of 200 kV. The oxidation state of the different chemical elements was investigated by X-ray photoelectron spectroscopy (XPS; Theta Probe, Thermo Scientific). The binding energy was calibrated from the C–C contribution due to the C 1s adventitious carbon signal at 284.8 eV. Data analysis was made with the CasaXPS software.^29^ The electrical stability was determined by exposure of the ITO/APC:O/ITO electrodes to 95% relative humidity and 90 °C for 330 h, while the film resistivities were measured every 12 h.

### 2.3 Optical Simulations

**Figure 1:**
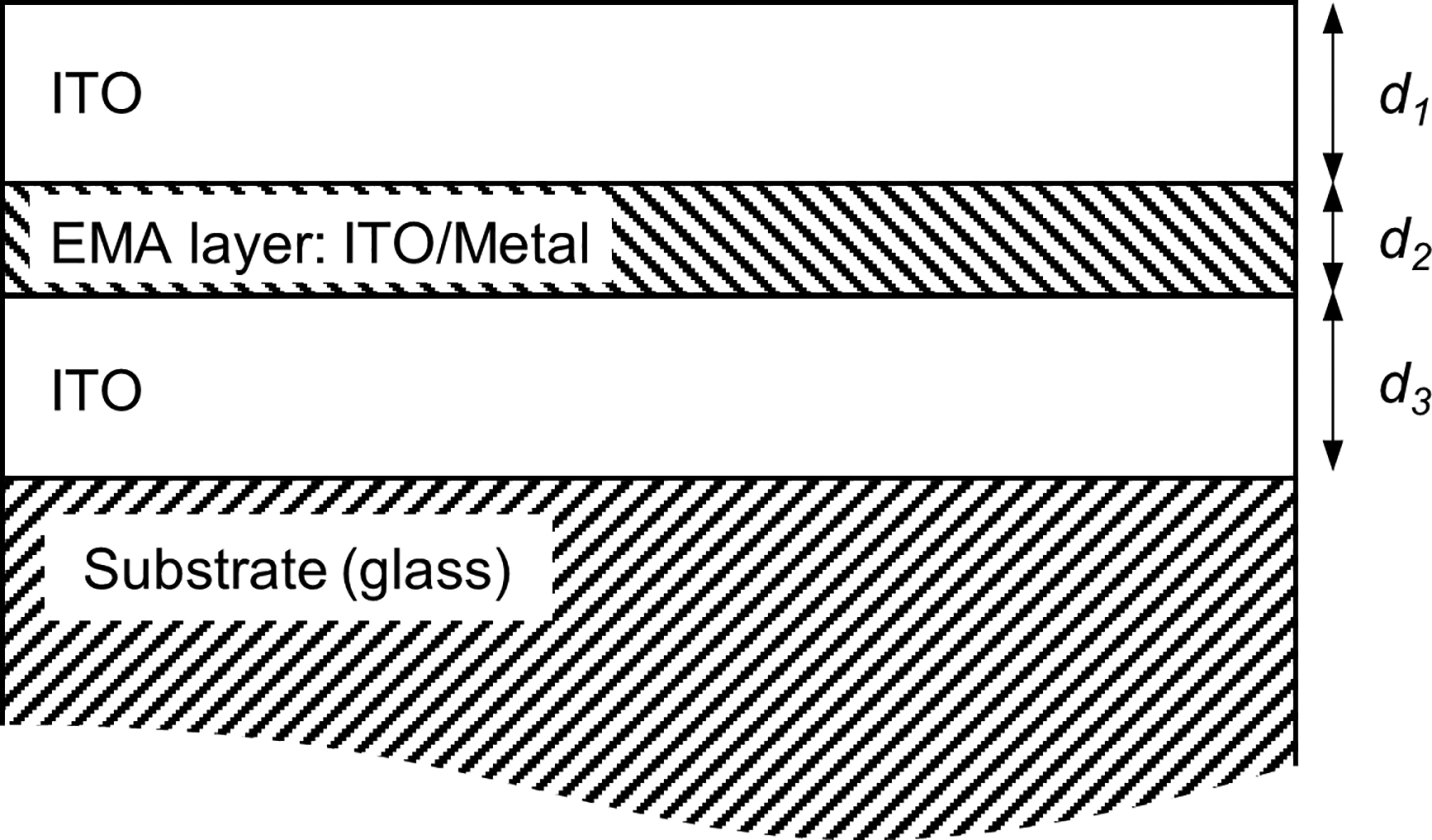
Layer stack used for effective medium approximation (EMA) modeling of the optical properties of the OMO structure.

The optical properties of the OMO coatings were modeled by using a four-layered model as depicted in Figure 1. The layer stack comprises a thin film of thickness *d*_2_ (*d*_2_ = 6.3 ± 1.2 nm) consisting of a mixed ITO/metal layer, sandwiched between two thin ITO layers of thicknesses *d*_1_ (*d*_1_ = 40.0 ± 4.9 nm) and *d*_3_ (*d*_3_ = 41.0 ± 3.6 nm). These three thin (optically coherent) layers are placed on a thick (optically incoherent) glass substrate (represented by a constant refractive index *n* = 1.52). The OMO structure thus constructed was modeled by the Bruggeman effective medium approximation (EMA),^30^ with a filling factor (volume fraction) *ff* of the metallic phase close to 1 (*ff* = 0.85 ± 0.09 nm). The Bruggeman approximation is used to include the roughness (inhomogeneity) of the metallic particulate layer. In the model fitting, parameters *d*_1_, *d*_2_, *d*_3_, and *ff* were not allowed to vary freely but in a fixed interval that was consistent with the experimental observations. In the case of the filling factor, *ff*, this interval was established qualitatively from the SEM and AFM observations. In the simulations, the optical properties of ITO were represented by a constant dielectric background ε_∞_ = 4 and a Drude oscillator model characterized by the plasma and relaxation frequencies Ω_p_, and Ω_τ_, respectively.^31^ For ITO, Ω_p_|_ITO_ = 5944.5 cm^–1^ and Ω_τ|ITO_ = 497.65 cm^–1^ were used and obtained by fitting the model to a single ITO 70 nm thick film (not shown). This set of Ω_p_|_ITO_ and Ω_τ|ITO_ parameters correspond to a free charge carrier concentration of 1.5 × 10^20^ cm^–3^ and a resistivity of 8.5 × 10^–4^ Ω m, in good agreement with Hall measurement data obtained for the single ITO film. Ω_p|ITO_ and Ω_τ|ITO_ corresponding to the ITO phase have been considered constant for all samples studied. Finally, the interband band gap absorption of ITO was modeled by a Tauc–Lorentz oscillator.^32^ The optical model was implemented in the commercial software Scout,^33^ and the parameters presented in Table S1 were obtained after fitting the model to the experimental data by using the downhill simplex method.^33^

## 3 Results and Discussion

**Figure 2:**
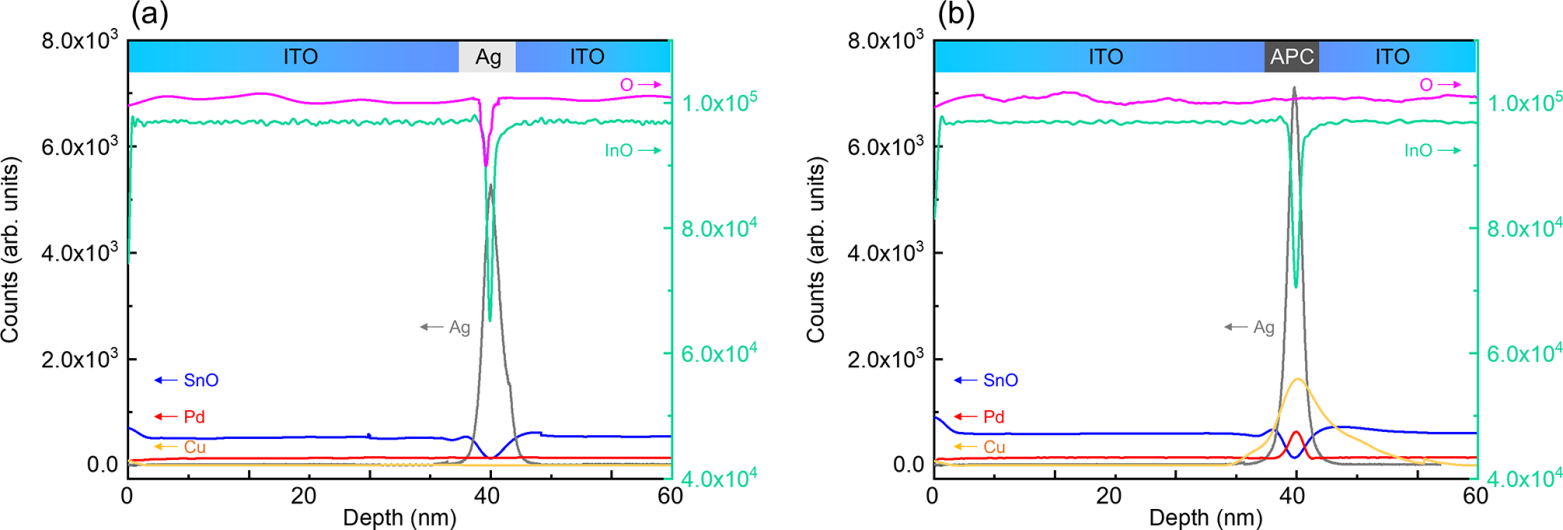
TOF-SIMS depth profiling results of (a) ITO/Ag/ITO and (b) ITO/APC:O/ITO at Γ = 3%. There is no significant difference in InO, SnO, and Ag; however, the oxygen content in the region on metal layer is significantly different.

Figure 2 shows depth profiling analysis obtained by TOF-SIMS corresponding to the samples ITO/Ag/ITO and ITO/APC:O (Γ = 3.0%)/ITO. The signal intensity corresponding to InO, SnO, and Ag as a function of film depth is practically the same in both cases, but not surprisingly, the O content distribution is different. In the case of the Ag-based OMO, the intensity of the O signal drops when the Ag layer is reached (Figure 2a). On the other hand, for the APC-based OMO, the O signal does not drop when the APC layer is reached (Figure 2b), confirming the incorporation of oxygen in the APC layer. The TOF-SIMS results in Figure 2 further prove the APC composition in the ITO/APC:O/ITO structure. We note in Figure 2b that small amounts of Cu migrate into adjacent ITO regions, thus further diluting the Cu concentration. Importantly, and as we shall see below, the small amounts of Cu present in ITO films do not deteriorate the optical or electrical properties of the ITO/APC:O/ITO film.

In Figure S1, XRD patterns of ITO/Ag/ITO and ITO/APC:O/ITO at various oxygen flow ratios, Γ, are presented. It is evident that the OMO films are amorphous. In general, for oxide–metal–oxide (OMO) structures, the top ITO layer readily crystallizes when it is deposited on a metal layer. In contrast, our metal layers with very small thicknesses, <10 nm, do not promote crystallization, in agreement with previous studies.^34^

**Figure 3:**
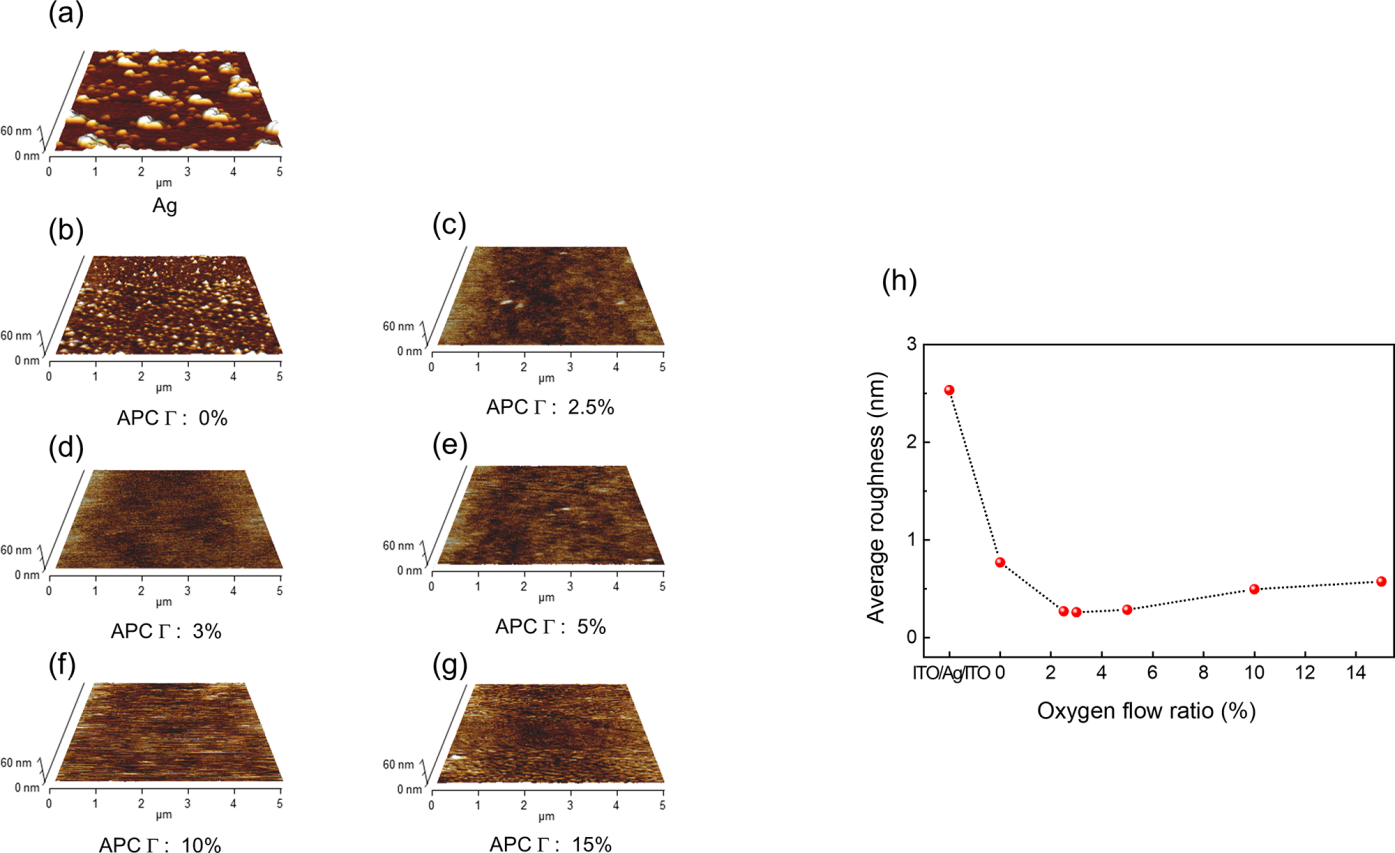
(a–g) AFM images of Ag and APC:O films prepared at different oxygen flow ratios and (h) corresponding plots of root-mean-square (rms) surface roughness obtained from lines scans of the AFM images. The decreased rms as a function of oxygen flow ratio is associated with suppression of Ag island agglomeration and formation of continuous APC:O layers.

**Figure 4:**
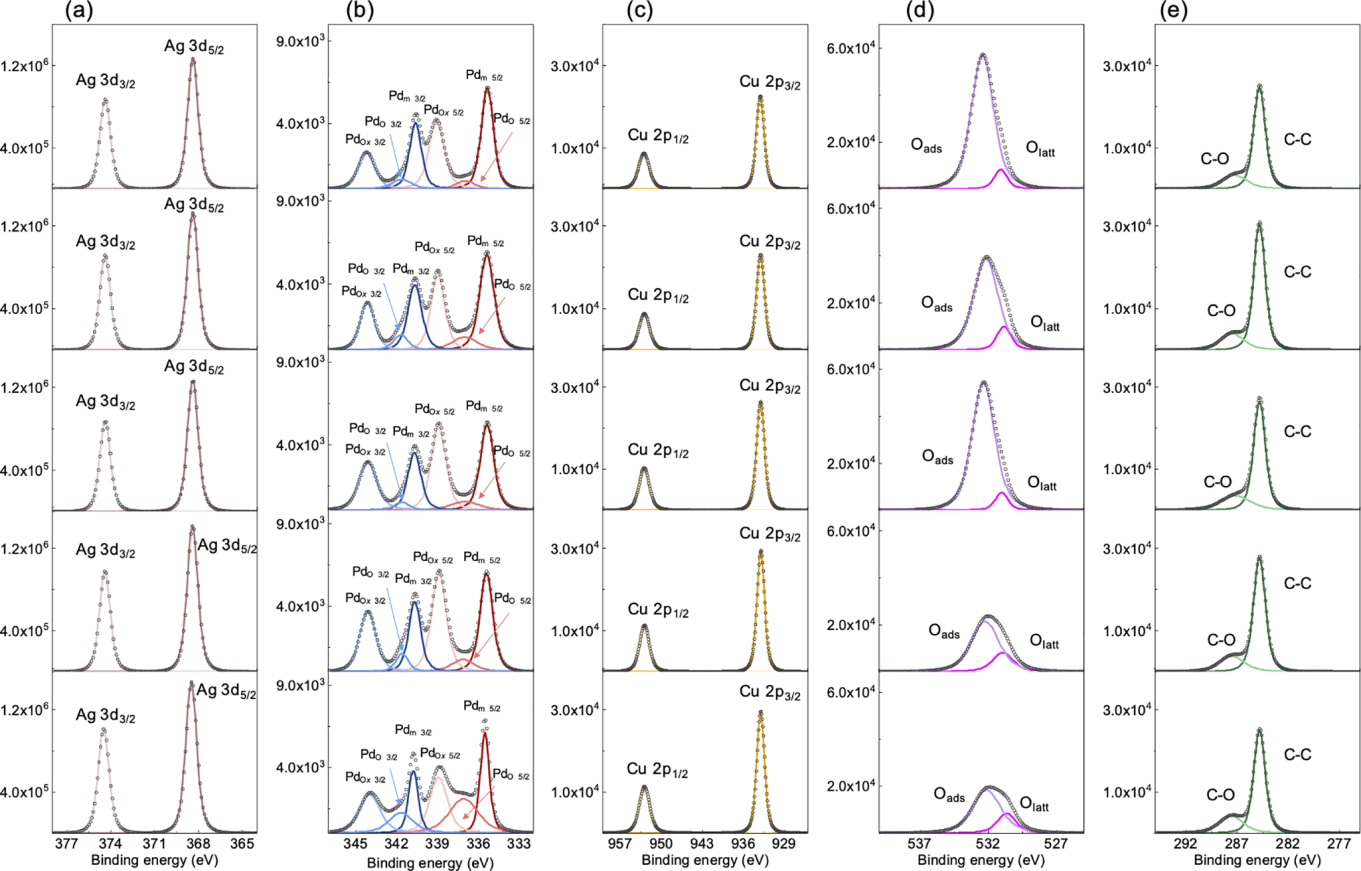
High-resolution XPS core–electron spectra of (a) Ag 3d, (b) Pd 3d, (c) Cu 2p, (d) O 1s, and (e) C 1s for APC:O films prepared at different oxygen flow ratios, from top to bottom: 0, 2.5, 3, 5, and 10%. The dashed curves show peak deconvoluted bands. There is no significant difference in Ag 3d, Cu 2p, and C 1s. However, the O 1s orbital peak clearly shows decrease of adsorbed oxygen (O_ads_ = 532.1 eV) and increase of lattice oxygen (O_latt_ = 531.3 eV) which indicates oxide formation. Pd oxidation related peak (Pd_O*x*5/2_ = 338.7 eV, Pd_O5/2_ = 337.1 eV, Pd_O*x*3/2_ = 343.8 eV, and Pd_O3/2_ = 341.7 eV) increased as a function of oxygen flow during the deposition process. PdO_*x*_ (*x* > 1) peaks appear at higher binding energy compared to PdO due to highly oxidized Pd atoms in nonstoichiometric Pd oxide.

Figures 3a–g depict AFM topographical images of 5 nm thick (nominal) Ag and APC:O single films deposited at different values of Γ. In addition, the average root-mean-square (rms) surface roughness, as obtained by AFM measurements, is presented for each set of samples in Figure 3h. As can be observed in Figure 3a, the Ag layer forms a particulate film consisting of Ag islands; i.e., the thickness of the Ag layer is below the percolation threshold, above which a continuous film can be achieved. This is not an unexpected result: the threshold thickness for achieving a continuous Ag thin film is known to be around 10 nm. Figure 3b shows that substitution of Ag by an APC alloy results in the formation of smoother, more compact film, while still exhibiting particulate features in AFM. In contrast, the APC:O films exhibit smooth morphologies with reduced rms. Still, as evident in Figure 3b, APC films obtained at Γ = 0% present a relatively high surface roughness and does not form continuous films, although the APC islands become considerable flatter than in the case of Ag, suggesting a relatively stronger support interaction. Irrespectively of Γ, APC:O films exhibit considerably lower surface roughness than pure Ag coatings. The formation of a continuous 5 nm thick film takes place above about Γ = 3% (Figure 3c–g). Increasing Γ up to 3% results in a dramatic drop of the surface roughness of the APC:O films down to rms = 0.26 nm (Figure 3h). We note the importance of oxygen for obtaining a continuous APC:O film. When Γ > 3%, a slightly increased surface roughness is observed in Figure 3e–g, which can be attributed to formation of Pd oxide nuclei, as seen in the XPS data (Figure 4). Thus, in the APC alloy thin film, Pd suppresses Ag agglomeration due to superficial Pd oxide formation, indicating decrease of the interface energy of the Ag alloy and stronger substrate interaction, thus promoting continuous layer growth. At too high oxygen loading, however, extensive oxide formation occurs, leading to not only some roughening but also deteriorating conductivity as discussed below.

Figure 4 shows high-resolution XPS spectra of APC:O thin films prepared at different oxygen flow ratios, Γ. No significant differences are observed in the Ag 3d, Cu 2p, and C 1s spectra as a function of O concentration in the films. However, according to the TOF-SIMS results presented in Figure 2, Cu partially diffuses into neighboring ITO regions. This, together with the fact that the initial (before diffusion) Cu content in the APC film is already small, can hinder the observation of Cu oxide species by XPS. In contrast, the Pd 3d spectrum clearly shows an increasing degree of Pd oxidation as Γ increases. In Figure 4 it is seen that PdO_*x*_ peaks appear at higher binding energies compared to PdO (Table S2), which we attribute to highly oxidized Pd atoms. This is similar to what has previously been reported for Pd^4+^ species in very small oxidized Pd nanoparticles,^35^ thus indicating the formation of a PdO_*x*_ phase with *x* > 1, containing mixed Pd^2+^ and Pd^4+^ in the ultrathin APC:O films. Notably, at small O concentration (Γ < 5%), mainly highly oxidized Pd^4+^ is observed, whereas at high O concentration Pd^2+^, associated with stoichiometric PdO oxide, starts to dominate. These results suggest that the nonstoichiometric PdO_*x*_ phase in the APC:O structure is responsible for the suppression of APC agglomeration and, as it will be discussed later, the good electrical properties observed in these samples. Confirming this interpretation, the O 1s orbital peak shows a relative decrease of adsorbed oxygen (O_ads_ = 532.1 eV) and increase of lattice oxygen (O_latt_ = 531.3 eV), demonstrating increased oxidation of Pd as a function of oxygen flow during the deposition process. This suggests that superficial PdO_*x*_ formation decreases the surface energy of the alloy and strengthen the substrate interaction, thus promoting the growth of a continuous APC:O single-layer thin film.

**Figure 5:**
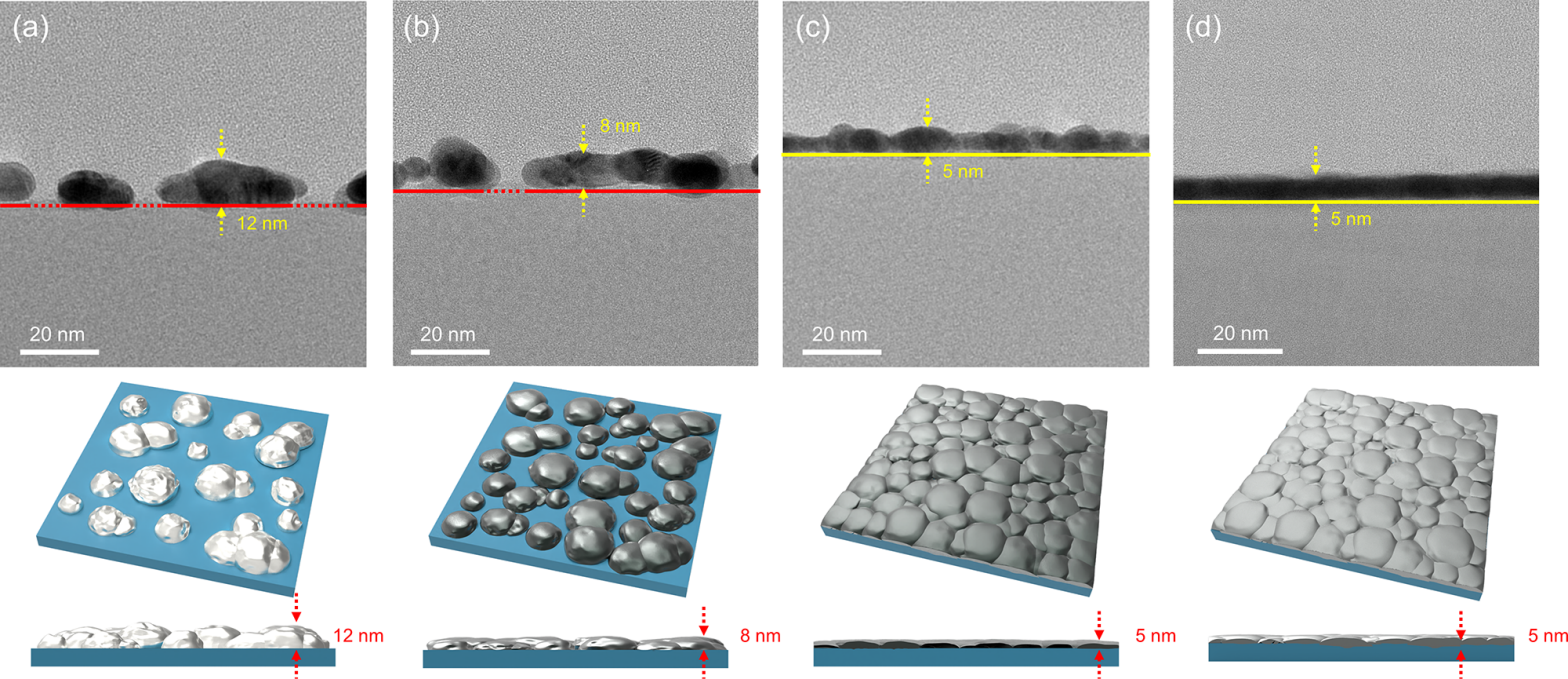
FE-TEM cross-sectional images of (a) Ag, (b) APC, (c) APC:O (Γ = 3%), and (d) APC:O (Γ = 10%) showing suppressed Ag agglomeration in the APC:O film structure. Lower figures depict schematic 3D and cross-sectional illustration of the FE-TEM images.

Figure 5 shows FE-TEM cross-sectional images of ITO/Ag/ITO, ITO/APC (Γ = 0%)/ITO ITO/APC:O (Γ = 3%)/ITO, and ITO/APC:O (Γ = 10%)/ITO structures of nominal thickness 5 nm deduced from the calibrated deposition growth rates. Each FE-TEM image in Figure 5 is accompanied by a schematic drawing for their easier interpretation (Figure 5, lower panels). The FE-TEM images confirm that the Ag layer consists of agglomerated Ag islands forming a particulate layer with thickness of about 12 nm. The same result is apparent for the APC alloy film (Γ = 0%), but as expected from the AFM analysis of the single film, in this case the particles are flattened out, forming an 8 nm thick particulate layer. In contrast, in the case of APC:O (Γ = 3%), little or no coalescence is observed, and a 5 nm thick continuous film is formed, showing that the oxygen-modified APC alloy suppresses metal particle agglomeration. Figure 5d shows the results for an APC:O (Γ = 10%) film. This result further shows that above about Γ = 3% agglomeration of APC:O is avoided due to Pd oxidation (Figure 4), yielding about the same film thickness (∼5 nm). Above 3% XPS data show that an excessive oxide layer forms at high oxygen concentration during synthesis, which also results in a slightly increased surface roughness, as shown by the AFM data (Figure 3).

**Figure 6:**
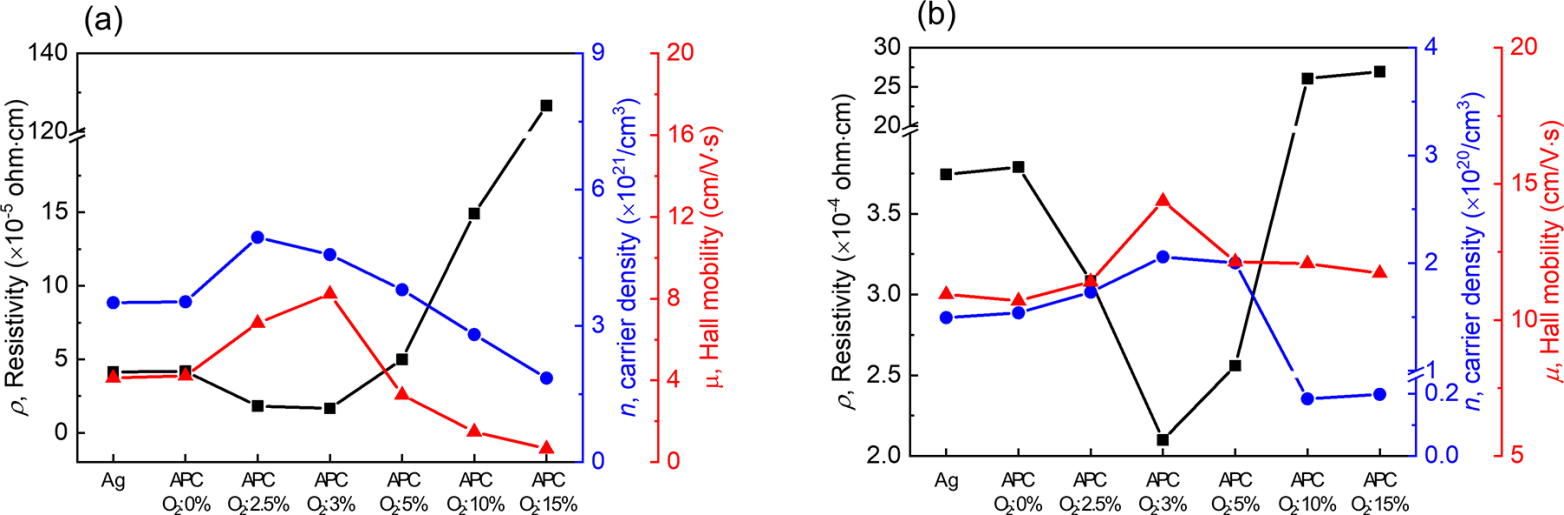
Electrical transport properties of (a) single Ag and APC metal layers with thickness of 5 nm and (b) ITO (40 nm)/Ag or APC:O/ITO (40 nm) as a function of oxygen flow ratio, Γ (see text).

Figure 6a shows the resistivity, ρ, carrier density, *n*, and Hall mobility, μ, corresponding to a single Ag layer as well as for various APC:O layers with different oxygen compositions, sputtered at different O_2_ partial pressures (different Γ values). All these films are expected to be 5 nm thick based on the calibrated sputtering growth rate. The Ag and APC films (the APC:O films deposited at Γ = 0%) exhibit similar electrical properties, as shown in Figure 6a. As Γ increases, ρ decreases, reaching a minimum at Γ = 3% coinciding with a remarkable increase of μ. As it will be demonstrated below, metallic layers deposited at Γ < 3% are still discontinuous, and hence the maximum value of μ measured when Γ = 3% is attributed to the formation of an APC:O film, transforming the structure to a connected network of grains. However, a further increase of Γ, above 3%, results in deteriorating electrical properties of the APC:O layer, which, as elaborated above, can be attributed to the formation of extensive Pd oxide and the concomitant drop of *n* and μ. Figure 6b shows ρ, *n*, and μ for the analogous OMO multilayered film structures ITO (40 nm)/Ag (5 nm)/ITO (40 nm) and ITO (40 nm)/APC:O (5 nm)/ITO (40 nm) with the APC:O layer deposited at different values of Γ. Results presented in Figure 6b and are consistent with the results shown in Figure 6a. The use of APC:O deposited at Γ = 3.0% results in a minimum of ρ. Again, this is attributed to an enhanced μ due to formation of a continuous metal film. Similarly, increasing Γ above 3% has a detrimental effect in ρ which can be explained by oxidation and drop of *n* and μ, as observed in Figure 5b. The increased resistance and lower mobility at Γ > 3% coincide with increased rms seen in Figure 3h, further supporting that bulk oxidation occurs at the highest Γ values. However, compared to a single metal layer, the OMO structure shows higher resilience of electrical transport properties toward oxidation, which can be attributed the protective embedding ITO.

The results above can be understood by oxide formation at the surface of APC. The thin film nucleation and growth mechanisms, which determine whether the thin film microstructure is particulate or continuous, depend on the cohesive energy of the metal atoms, their surface energy, and the binding energy to the supporting ITO surface.^36,37^ It is known that Ag films become rough at small thicknesses due to the large interface energy of Ag favoring Volmer–Weber growth. Both Pd and Cu can alloy with bulk Ag, and it is known that Pd alloyed with Ag (as in APC) can suppress agglomeration. By use of small amounts of Pd addition in Ag, thin Ag coatings have been achieved in the past.^25,27,38,39^ Our results show that purposefully adding small amounts of oxygen in the in the plasma during sputtering of APC, up to about Γ = 3%, promotes formation a superficial PdO_*x*_ phase that prevents particle agglomeration in a controlled way leading to ultrathin, continuous, electrically conducting APC:O films. In contrast, too high Γ values lead to extensive PdO formation evident in Figure 4, with an increased surface roughness (Figure 3) and deteriorating electrical properties (Figure 6). As shown below, APC:O also shows resilience to oxidation by ambient humidity and elevated temperatures.

**Figure 7:**
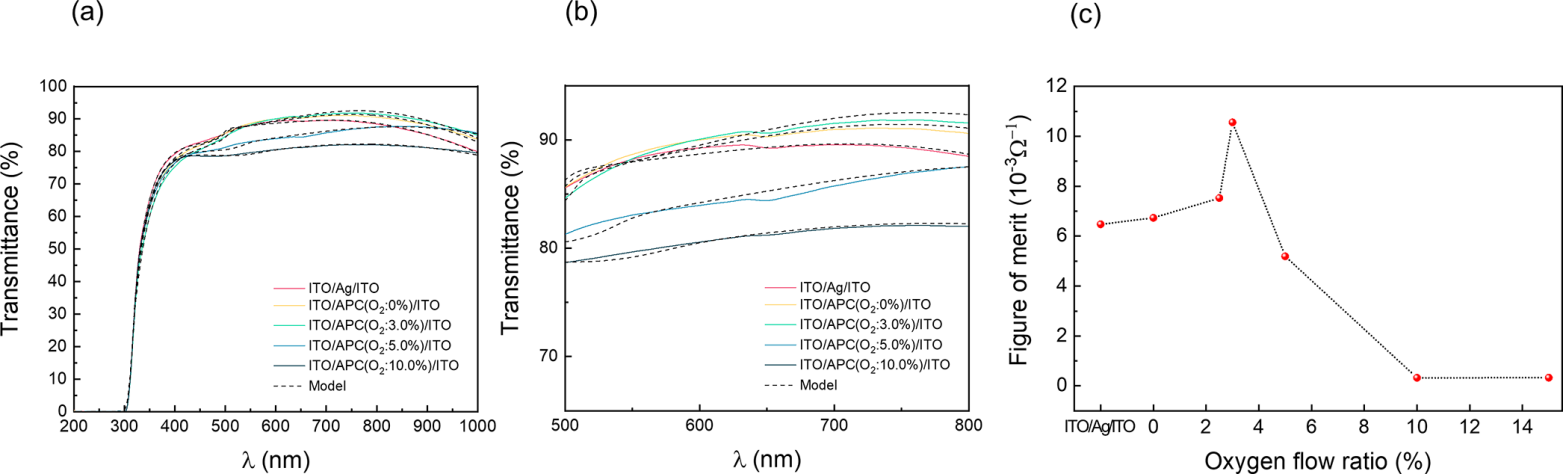
(a) Measured transmittance spectra for ITO/Ag/ITO and ITO/APC:O/ITO deposited under different oxygen to Ar ratio, Γ (color lines), and calculated transmittance spectra using the Bruggeman effective medium approximation (see text). (b) Magnification of (a) in the region between 500 and 800 nm. (c) Figure of merit (FoM, *T*^10^/*R*_sheet_) of ITO/Ag/ITO and ITO/APC:O/ITO as a function of Γ is plotted.

Figure 7a shows experimental and calculated optical transmittance, *T*, corresponding to ITO/Ag/ITO and ITO/APC:O/ITO prepared at different Γ ratios. Figure 7b shows a magnification of the visible part of the spectrum of the curves presented in Figure 7a. Calculated curves were obtained by using the three-layered model described in section 2.2. Transmittance curves were found to be in good agreement with the experimental data for both ITO/Ag/ITO and ITO/APC (Γ = 0)/ITO (Figure S2). The metallic layer was here modeled by using the optical constants of Ag reported by Johnson and Christy.^40^ This confirms that Ag thin films retain practically the same refractive index of Ag bulk even for very low thicknesses.^41^ However, using the refractive index of Ag fails when modeling ITO/APC:O/ITO coatings deposited at Γ > 0%. Instead, the metallic layer must be represented by a Drude oscillator model with fitting parameters Ω_p|Metal_, and Ω_τ|Metal_, as specified for each sample in Table S1. The parameters in Table S1 give results that are in excellent agreement with the experimental data, as shown in Figure 7. Note that for Γ = 0 the parameters Ω_p|Metal_, and Ω_τ|Metal_ presented in Table S1 also show agreement with those reported for Ag.^40^ As shown in Figure 6b, *T* decreases for Γ > 3%. According to the data presented in Table S1, the lower transparency in the visible region of the OMO coatings deposited at Γ > 3% can be attributed to a decreased mobility of the free electrons in the metallic layer, i.e., an increase of Ω_τ|Metal_. This may be attributed to scattering by impurities associated with the incipient formation of a disordered oxide phase.^42^ We note that despite the success of our optical model in reproducing the optical properties of the different OMO coatings, the conclusions drawn from the modeling should be regarded with some caution. In particular, multipole interactions with the surrounding ITO, although not a metallic conductor, are disregarded.^30^ A more complete treatment requires details about the particular geometry of the ITO/metal interface and explicit treatment of their mutual field-induced polarization. Figure 7c shows the figure of merit, FoM, calculated according to the Haackes’s formula FoM = [*T*(λ = 550 nm)]^10^/*R*_sheet_,^43^ where *R*_sheet_ is the sheet resistance of the OMO coating presented in Figure 6. The OMO coating ITO/APC:O (Γ = 3.0%)/ITO exhibits the highest FoM thanks to its excellent Hall mobility, typically for a continuous APC thin film layer without percolation, combined with high *T* values in the visible region.

**Figure 8:**
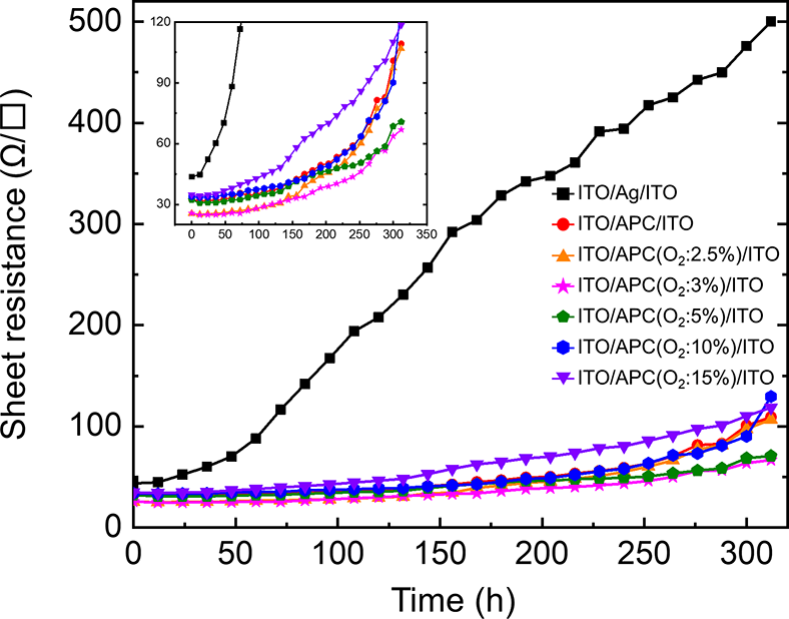
Change of sheet resistance as a function of time for ITO/Ag/ITO and ITO/APC:O/ITO (Γ = 3%) in relative humidity 95% and 90 °C. The inset shows an enlargement of the ordinate axis to facilitate comparisons of the ITO/APC:O/ITO films.

Figure 8 shows the sheet resistance as a function of time for ITO/Ag/ITO and for ITO/APC:O/ITO samples deposited at Γ values ranging from 0% to 15%. The samples were subjected to high humidity (95%) and temperature (90 °C) conditions for 330 h, and their sheet resistance was intermittently measured. When Ag is used in the OMO, the sheet resistance increases rapidly as a function of time. On the other hand, when the APC alloy is used, the OMO shows much better electrical stability. According to these results, it is possible to assume that high humidity and temperature cause agglomeration of Ag which reduces the electrical conductivity. On the other hand, the use of APC results in an excellent electrical stability, which can be attributed to the suppression of agglomeration by the presence of Pd and Cu and the formation of superficial Pd oxide. Table S3 shows comparisons of the electrical and optical properties of the APC:O films reported here and previously published data.

**Figure 9:**
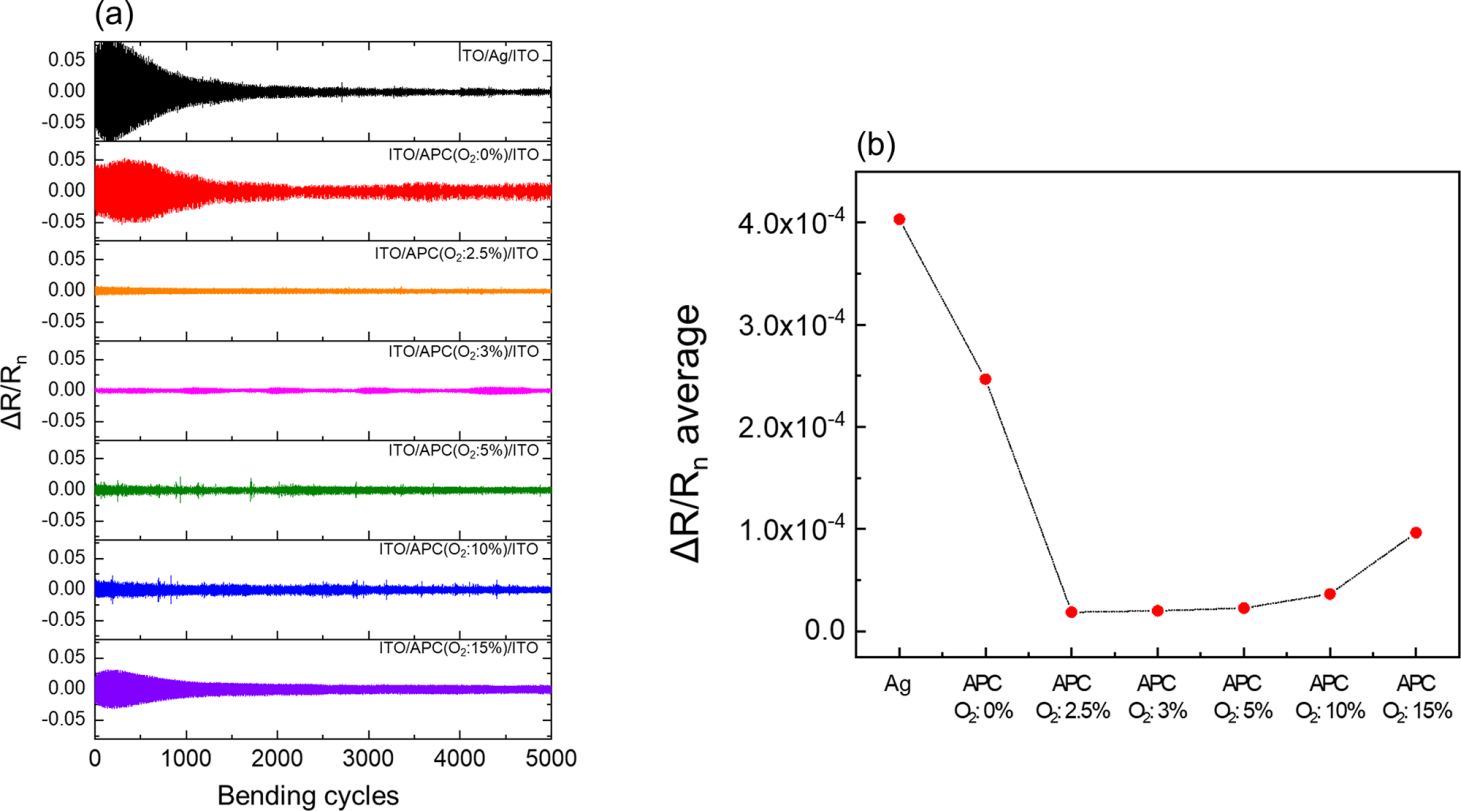
Dynamic bending test results employing a bending radius of 5 mm of ITO/Ag/ITO and ITO/APC:O/ITO deposited under different oxygen-to-Ar ratio, Γ. (a) shows Δ*R*/*R*_*n*_ as a function of cycling number, and (b) shows the average resistance change in (a). The duration for each cycle is 2 s, with 0.5 s interval between each measuring. Δ*R* is determined to be *R*_*n*+1_ – *R*_*n*_.

Finally, in Figure 9 we present the results from dynamic bending test results employing a bending radius of 5 mm. The duration for each cycle is 2 s, with 0.5 s interval between each measuring. The tests were conducted for 5000 cycles. The change in resistance, Δ*R*, was determined to be Δ*R* = *R*_*n*+1_ – *R*_*n*_. Figure 9a shows Δ*R*/*R*_*n*_ as a function of cycling number. Figure 9b shows the average resistance changes in Figure 9a as a function of Γ. It is evident that the well-known OMO structure ITO/APC(Γ = 0%)/ITO only shows slightly improved mechanical stability compared to ITO/Ag/ITO. However, the ITO/APC:O/ITO obtained by controlled addition of oxygen in the film synthesis exhibit dramatically improved mechanical stability, with an optimum coinciding with the continuous, flat and electrically well-conducting APC:O film at Γ = 3%. Above Γ > 3%, Δ*R*/*R*_*n*_ slightly increases because of oxide formation, making it brittle. In Figure S3 photographs of ITO/Ag/ITO and ITO/APC:O/ITO thin films on PET are shown. All samples exhibited larger than 80% transmittance in visible light region.

## 4 Conclusions

It is shown that ultrathin (5 nm thick) and continuous oxygen-containing Ag–Pd–Cu metallic films (APC:O) can be made by reactive magnetron sputtering deposition on flexible PET substrates using a single Ag–Pd–Cu target and an oxygen-containing argon plasma. Such films can be embedded between two ITO films to obtain oxide–metal–oxide (OMO) TCM coatings in the configuration ITO/APC:O/ITO, which exhibit superior electrical, optical, corrosion stability, and mechanical bending properties. The reason for the improved optoelectronic properties is the suppression of metal particle agglomeration in the APC:O layer due to superficial Pd oxidation in the APC alloy. While Ag thin films with nominal thickness of 5 nm agglomerate, APC:O films yield smooth, continuous ultrathin films in the 5 nm range, approaching single-layer films. The APC:O films exhibit low resistivity, high Hall mobility, and excellent optical transparency. Optimal APC:O films were obtained for argon-to-oxygen ratio Γ ≈ 3%. Above Γ > 5.0%, the properties of the optical and electrical properties of the APC:O films deteriorate due to extensive PdO nucleation yielding coarser films. The combined AFM, FE-TEM, and electrical transport data show that oxygen incorporation in the APC alloy suppress agglomeration, suggesting a synergetic effect of the different elements present in the APC alloy as well as the controlled surface Pd oxidation. Both ITO/Ag/ITO and ITO/APC:O/ITO structures were subjected to high relative humidity/high temperature and mechanical bending tests. While the Ag-based OMO coatings subjected to humidity tests suffered from dramatic increase of sheet resistance attributed to oxidation and agglomeration of Ag, the ITO/APC/ITO structures showed remarkable stable sheet resistance. The same results were obtained from the bending tests. In all cases ITO/APC:O/ITO films with Γ = 3.0% showed the best results, demonstrating a balance between deep oxidation and superficial surface oxidation. We conjecture that ITO/APC:O/ITO coatings may find important applications as TCMs in optoelectronic applications, in particular where mechanical flexibility is required.

## Supporting Information Available

Drude oscillator parameters Ω_p|Metal_ and Ω_τ|Metal_ for the optical modeling of the metallic layer; binding energy and peak fitting area of the Pd 3d region from XPS analysis; comparisons of electrical and optical performances with our results and previously published data; XRD diffractograms of ITO/Ag/ITO and ITO/APC:O/ITO as a function of oxygen flow ratio; measured and modeled transmittance curves for ITO/Ag/ITO and ITO/APC/ITO; photographs of ITO/APC:O/ITO thin films on a PET substrate (PDF)
