# Supplementary material for: Embedded Oxidized Ag–Pd–Cu Ultrathin Metal Alloy Film Prepared at Low Temperature with Excellent Electronic, Optical, and Mechanical Properties
Source: ACS Appl Mater Interfaces. 2022 Mar 22;14(13):15756–64. doi: 10.1021/acsami.1c23766 (PMC8990516; doi:10.1021/acsami.1c23766)
Supplement: Supplementary file 1 — am1c23766_si_001.pdf [file am1c23766_si_001.pdf]

Supporting Information

Embedded Oxidized Ag–Pd–Cu Ultra-Thin Metal Alloy Film Prepared at Low Temperature with Excellent Electronic, Optical and Mechanical Properties

Seohan Kim,[1,2] Jose Montero,[2] Janghee Yoon,[3] Yunju Choi,[3] Sungmin Park,[4] Pungkeun Song[4,*] and Lars Österlund[2,*]

[1]Material Technology Research Institute, Pusan National University, Busan 46241, Korea

[2]Department of Materials Science and Engineering, The Ångström Laboratory, Uppsala University, P. O. Box 35, SE-75103, Uppsala, Sweden

[3]Busan center, Korea Basic Science Institute, Busan 46742, Korea

[4]Department of Materials Science and Engineering, Pusan National University, Busan 46241, Korea

* E-mail: pksong@pusan.ac.kr, lars.osterlund@angstrom.uu.se

Table S1 Drude oscillator parameters $\Omega_{p|Metal}$, and $\Omega_{\tau|Metal}$ corresponding to the metallic layer, i.e., Ag or APC:O at various sputtering oxygen/Ar gas mixing ratios, Γ.

Sample	$\Omega_{p|Metal}$(cm^{-1})	$\Omega_{\tau|Metal}$ (cm^{-1})
ITO/Ag/ITO	75689	320
ITO/APC(Γ=0)/ITO	68946	307
ITO/APC(Γ=3)/ITO	71306	202
ITO/APC(Γ=5)/ITO	75195	943
ITO/APC(Γ=10)/ITO	71460	2231

Table S2 Binding energy and peak fitting area of Pd 3d spectra of APC thin films prepared with oxygen flow ratios.

	Pd $3d_{m\ 5/2}$	Pd $3d_{O\ 5/2}$	Pd $3d_{O\ sub\ 5/2}$	Pd $3d_{m\ 3/2}$	Pd $3d_{O\ 3/2}$	Pd $3d_{O\ sub\ 3/2}$
Binding energy (eV)	335.6	339.0	337.1	340.8	344.2	341.7

Oxygen flow ratio (%)	O_{ads}	O_{latt}	Pd $3d_{m\ 5/2}$	Pd $3d_{O\ 5/2}$	Pd $3d_{Osub\ 5/2}$	Pd $3d_{m\ 3/2}$	Pd $3d_{O\ 3/2}$	Pd $3d_{O\ sub\ 3/2}$	Sum Pd 3d / O 1s	$Pd_{m\ 5/2}$ / $Pd_{m\ 3/2}$	$Pd_{O\ 5/2}$ / $Pd_{O\ 3/2}$
0	129880	10886	7658	6374	891	4899	3790	1128	0.18	1.56	1.48
2.5	97388	14435	8489	6396	1993	5602	4298	1356	0.25	1.52	1.48
3	126594	9582	7185	7649	1482	4728	5395	699	0.20	1.52	1.50
5	60224	16828	7863	8953	1703	5255	6095	1370	0.41	1.50	1.43
10	52827	15748	5724	6136	6127	3762	4945	3367	0.44	1.52	1.48

Table S3 Comparison of electrical and optical performances of the ITO, APC:O and ITO/APC:O/ITO films reported in this study and prefiously published work.

Sample	Resistivity (Ω·cm)	Carrier concentration ($/cm^3$)	Hall mobility (cm/V·s)	Sheet resistance (Ω/□)	Transmittance (@550nm, %)
ITO 40 nm	5.817×10^{-4}	2.476×10^{20}	43.3	145.4	-
ITO 80 nm	4.385×10^{-4}	3.511×10^{20}	40.05	54.8	-
APC:O	1.66×10^{-4}	4.571×10^{21}	8.227	33.2	-
ITO/APC:O(5nm)/ITO	2.1×10^{-4}	2.058×10^{20}	14.37	24.7	88
ITO 50nm[7]	4.55×10^{-4}	2.66×10^{20}	52.74	91	-
ITO/Ag(4nm)/ITO[32]	-	-	-	68	65
ITO/APC(4nm)/ITO[32]	-	-	-	32	80

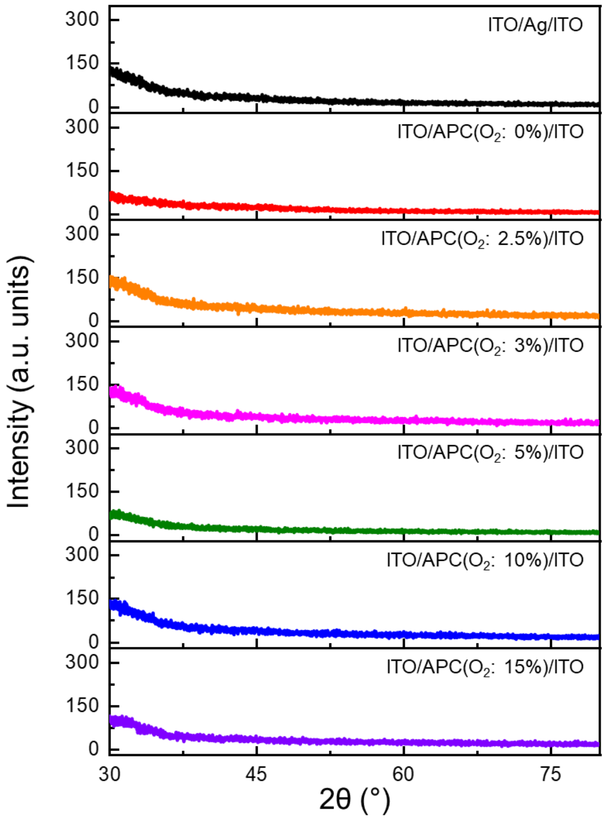

Figure S1 XRD diffractograms of ITO/Ag or APC:O/ITO as function of oxygen flow ratio, Γ.

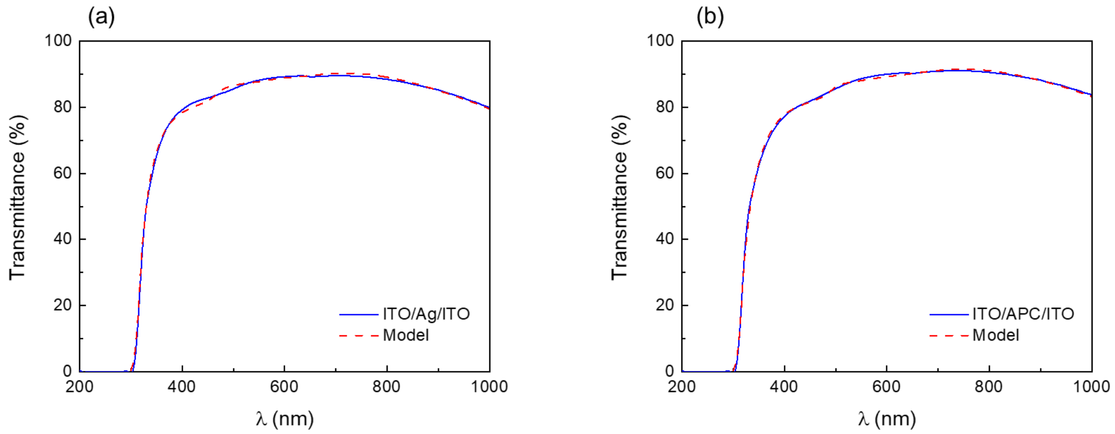

Figure S2 Transmittance curves for (a) ITO/Ag/ITO and (b) ITO/APC(Γ=0)/ITO. The plots show experimental and calculated results using the refractive index for Ag as reported by Johnson[37].

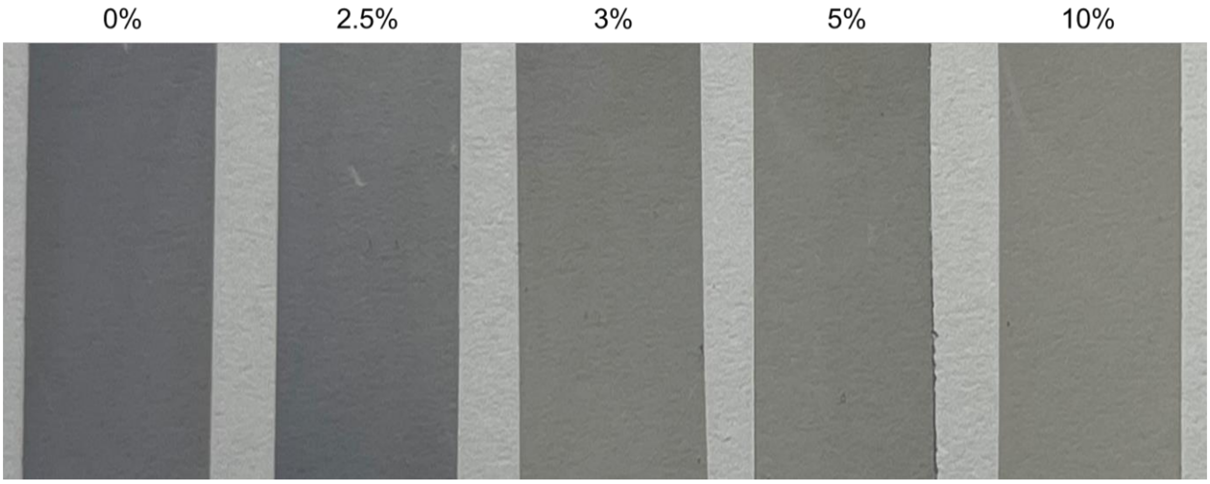

Figure S3 Photographs of ITO/APC:O/ITO thin films on PET substrate. The ITO/APC:O (0 % and 2.5%)/ITO appears slightly darker.
